# Eco-friendly Fluorescent Sensor for Sensitive and Selective Detection of Zn^2+^ and Fe^3+^ Ions: Applications in Human Hair Samples

**DOI:** 10.1007/s10895-024-03798-3

**Published:** 2024-07-03

**Authors:** Rasha M. Kamel, Sahar S. El-Sakka, Maram M. A. Abbas, M. H. A. Soliman

**Affiliations:** Chemistry Department, Faculty of Science, Suez University, Suez, 43518 Egypt

**Keywords:** Zinc, Detection, Fluorescence, Sensor, Iron, Pollution

## Abstract

A new eco-friendly sensor, 3-((6-((4-chlorobenzylidene)amino)pyridin-2-yl)imino)indolin-2-one (*CBAPI*) was synthesized and well characterized. The *CBAPI* sensor was employed for detecting Zn^2+^ and Fe^3+^ ions. It exhibited a low limit of detection at pH 6.0, with values of 2.90, for Zn^2+^ and 3.59 nmol L^−1^ for Fe^3+^ ions. The sensor demonstrated high selectivity over other interfering cations. Additionally, the high binding constants reflect the great affinity of sensor towards Zn^2+^ and Fe^3+^ ions. To further validate its quantification ability for Zn^2+^ ions, the synthesized *CBAPI* sensor was used to determine Zn levels in human hair samples, and the results were confirmed using atomic absorption spectroscopy (AAS). The AGREE metric tool was used to assess the method’s environmental impact and practical applicability. These positive outcomes indicated that the new method for detecting Zn^2+^ and Fe^3+^ ions is environmentally friendly and safe for humans. The developed *CBAPI* sensor represents a potential development in metal ion detection, combining sensitivity, selectivity, and rapidity.

**Supplementary Information:**

The online version contains supplementary material available at 10.1007/s10895-024-03798-3.

## Introduction

Industrial activities over the past century have significantly increased human exposure to heavy metals, whether through water, air, or food. This exposure can lead to acute or chronic poisoning. Heavy metals accumulate in the body, causing various toxic effects on tissues and organs. Additionally, they interfere with cellular processes such as growth, differentiation, and apoptosis [1].

Several heavy metals lack any known physiological benefits for humans. Lead, mercury, and cadmium are prime examples of such toxic metals. However, other metals are essential to human biochemical processes. For instance, zinc and iron serve as important cofactors for several enzymatic reactions in the human body [2].

Zinc, a trace element found in both human and natural environments, serves crucial roles in different biological processes. It is essential for normal growth and reproduction in higher plants, animals, and humans. Additionally, it contributes significantly to physiological growth and fulfils immune functions. Zinc is vital for the functionality of more than 300 enzymes, DNA stabilization, and gene expression [3, 4]. Similarly, iron is an essential element for nearly all living organisms. Due to its ability to exist in one of two oxidation states, it plays a vital role in diverse metabolic processes like oxygen transport, DNA synthesis, and electron transport. However, maintaining precise iron levels in body tissues is crucial because excessive iron can cause tissue damage by promoting the formation of free radicals [5, 6].

Various methods are employed for detecting Zn^2^⁺ and Fe^3^⁺ ions, including atomic absorption spectroscopy, inductively coupled plasma (ICP), and electrochemical techniques [7–20]. While these methods offer high sensitivity and selectivity, they often require costly equipment, complicated procedures, and time-intensive processes. In contrast, optical sensors provide valuable tools for precisely identifying clinical, chemical, and environmental species [21, 22]. Fluorescent sensors offer rapid and sensitive characterization of different species in samples. This technique stands out for its high sensitivity, specificity, simplicity, and cost-effectiveness compared to other analytical tools. It finds widespread use in environmental monitoring, industrial processes, medical diagnostics, forensics, and genetic analysis. Fluorescent sensors are applicable for both quantitative and qualitative analysis [23].

Our ongoing research focuses on creating chemosensors capable of detecting a range of environmentally and biologically relevant species [24–34]. In this study, we synthesized a novel fluorescent sensor, 3-((6-((4-chlorobenzylidene) amino) pyridin-2-yl)imino)indolin-2-one (*CBAPI*). This sensor has been successfully applied for the selective and sensitive detection of Zn^2^⁺ and Fe^3^⁺ ions.

## Experiments

### Materials and Reagents

Zinc nitrate Zn(NO_3_)_2_.6H_2_O, Ferric nitrate Fe(NO_3_)_3_.9H_2_O, and organic solvents were provided by Sigma Aldrich used as analytical grade with purity of ≥ 99.0%. all cationic compounds salts of Ag^+^, Al^3+^, Cu^2+^, Co^2+^, Hg^2+^, Ni^2+^, and Pd^2+^ were purchased from Aldrich.

### Instruments

Utilizing A Shimadzu-UV Probe Version 2.33 spectrophotometer, and JASCO FP-8300 Fluorescence Spectrophotometer.

### Methods

#### Solution Preparations

By dissolving 0.0336 g of *CBAPI* sensor in a 100 mL DMF to get a 1.0 × 10^−3^ mol L^−1^ stock solution. 0.0297 g and 0.0404 g of Zn(NO_3_)_2_.6H_2_O and Fe(NO_3_)_3_.9H_2_O, respectively, were dissolved in 100 mL bi-distilled water get a 1.0 × 10^−3^ mol L^−1^ stock solutions. The working solutions were prepared by diluting stock solutions to a known volume with bi-distilled water. Buffer solutions with pH values of 2.0, 4.0, 6.0, 8.0, and 10.0 were used.

#### Synthesis of 3-((6-aminopyridin-2-yl)Imino)Indolin-2-one (3)

To a solution of 2,6-diaminopyridine **(1)** (0.005 mol) in ethanol (20 mL), isatin3**(2)** (0.005 mol) was added. The reaction mixture was heated under reflux for 2 h, the solid compound **(3)** was filtered after cooling, washed with ethanol and used directly in the next reaction.

#### Synthesis of 3-((6-((4-Chlorobenzylidene)amino)Pyridin-2-yl)Imino)Indolin-2-one (4)* (CBAPI)*

A mixture of 3-((6-aminopyridin-2-yl)imino)indolin-2-one **(3)** (0.005 mol) and 4-chlorobenzaldehyde (0.005 mol) in butanol (20 mL) was heated under reflux for 4 h, the reaction was then cooled, the solid product obtained after cooling was collected by filtration and recrystallized from butanol to give **(4)** as red crystals. (yield 72%, m.p. over 300 °C. FTIR (ATR, Ʋ, cm^−1^): 3365 (NH), 1720 (CO), 1608 (C = N). ^1^H NMR (500 MHz, DMSO-*d*_6_) δ 10.13 (s, 1H), 9.96 (s, 1H), 7.93–7.87 (m, 2H), 7.57–7.50 (m, 2H), 7.46 (d, *J* = 7.1 Hz, 2H), 7.05–6.99 (m, 3H), 6.87 (d, *J* = 7.8 Hz, 2H).

#### Investigating the Impact of Zinc and Iron on CBAPI Fluorescence

0.2 mL of *CBAPI* sensor (2.0 × 10^–5^ mol L^−1^) solution and 4 mL of buffer solution of pH 6.0 were transferred to a 1 cm quartz cell, and successive additions of Zn^2+^ (0.00– 9.09 × 10^–5^ mol L^−1^ ppm) solution or Fe^3+^ (0.00– 3.54 × 10^–5^ mol L^−1^ ppm) solution were added. Within a few seconds of the addition of different analyte concentrations (Zn^2+^ or Fe^3+^ ions), the emission intensity measurements were monitored, and a calibration curve was developed, revealing an emission signal assigned at λ_em_ = 408 nm after applying excitation at λ_ex_ 335 nm.

#### Assaying Zinc in Human Hair Samples

One gram of hair was cut using scissor to pieces of less than 1 cm, then washed using bi-distilled water, then methanol, and left to dry in the air at room temperature. 12 mL of 90% nitric acid was added to dry hair and kept at room temperature for 10 min, then 1 ml of concentrated perchloric acid was added and heated on a hot plate at 200 °C for 1 h. until brown fumes converted into dense white fumes [35]. The clear solution was allowed to cool and dilute to 25 mL with bi-distilled water, and then analyzed using atomic absorption spectroscopy.

## Results and Discussion

### Characterization of CBAPI Sensor

**Scheme 1:**
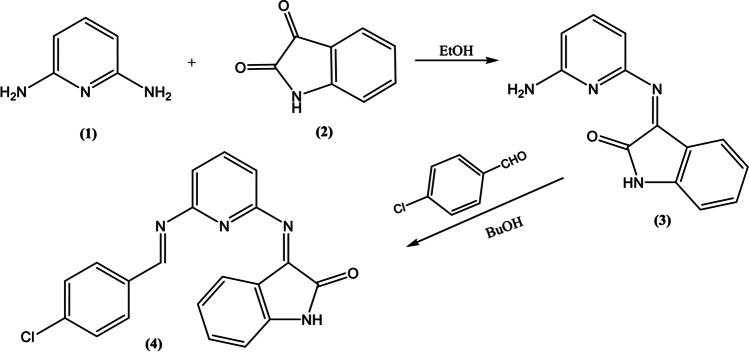
The synthesis mechanism of *CBAPI* sensor

The starting 3-((6-aminopyridin-2-yl) imino) indolin-2-one **(3)**, was synthesized by the condensation reaction of 2,6-diaminopyridine **(1)** with isatin **(2)**. Subsequent condensation of **(3)** with 4-chlorobenzaldehyde afforded the target Schiff base **(4) (**Scheme 1**)**.

**Fig. 1:**
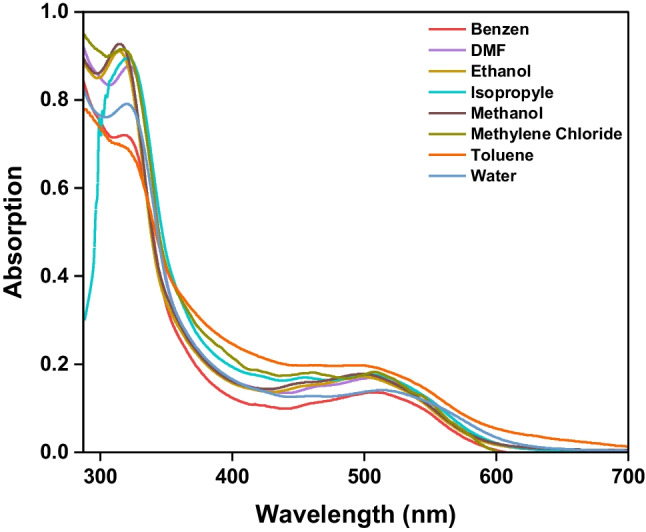
UV absorption spectra for 1 × 10^–4^ mol L^−1^*CBAPI* sensor in different solvents

The proposed structure of **(4)***CBAPI* sensor was confirmed from spectral data. The IR spectrum showed absorption band at 3365 cm^−1^ which may be assigned due to *ν*(N–H) stretching of the indoline ring. A strong absorption band was observed at 1720 cm^−1^ indicating the presence of *ν*(C = O) group of isatin. The IR spectrum also revealed the presence of absorption band corresponding to the* ν*(C = N) at 1608 cm^−1 ^(SI: Fig. 1).

**Fig. 2:**
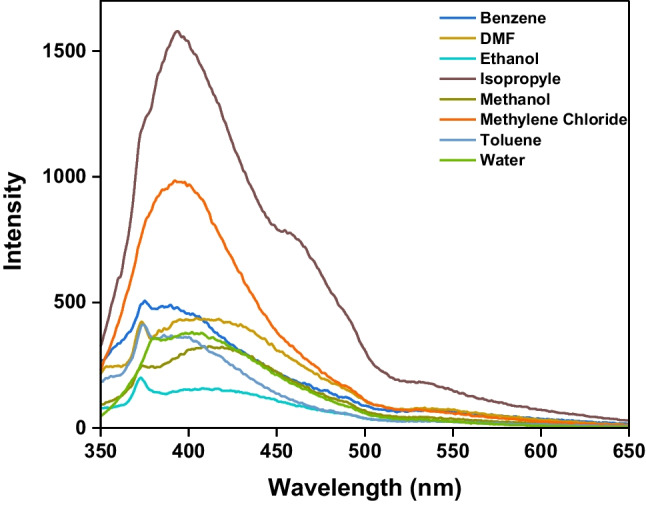
Emission spectra for 2 × 10^–5^ mol L^−1^*CBAPI* sensor at different solvents with λ_exc_ 335 nm

The ^1^H-NMR spectrum of compound **(4)** showed a broad singlet at 10.13 ppm which may be assigned due to proton of the NH group of indoline ring. The signal assigned to the azomethine proton was observed at 9.96 ppm as singlet signal. Because of the many J values and overlapping signals, the signals of the aromatic protons were observed as complex multiplets at around δ 6.87–7.93 ppm (SI: Fig. 2).

### Effect of Solvent on Absorption and Emission Spectrum of the CBAPI Sensor

Figure 1 illustrates the UV − vis spectra of 1 × 10^–4^ mol L^−1^*CBAPI* sensor in different solvents (ethanol, methanol, water, DMF, isopropyl alcohol, benzene, methylene chloride, cyclohexane, and toluene) (1:99) (v/v) DMF: solvent and the corresponding spectroscopic parameters are collected in Table 1. The emission spectrum of *CBAPI* sensor in different solvents at λ_exc_ 335 nm was depicted in Fig. 2.

**Table 1:** Spectral and photophysical parameters of *CBAPI* sensor in different solvents

Solvent	λ_abs_ (nm)	λ_exc_ (nm)	λ_em_ (nm)	ε (mol^−1^ cm^−1^ L) × 10^3^	Stock shift (cm^−1^) Δʋ = ʋ_exc_—ʋ_em_
Methanol	315, 501	335	411	9.27, 1.78	5519.8
Ethanol	316, 501	335	416	9.10, 1.72	5812.3
Water	321, 517	335	402	7.91, 1.41	4975.1
Isopropyl	321, 506	335	394	8.96, 1.77	4470.0
DMF	322, 507	335	417	8.77, 1.69	5869.9
Toluene	314, 501	335	371	7.01, 1.97	2896.6
Methylene Chloride	315, 463	335	396	9.16, 1.81	4598.2
Benzene	319, 511	335	375	7.20, 1.36	3184.1

The absorption spectra of the *CBAPI* sensor exhibited negligible changes with changing solvent polarity. Two absorption peaks occur at approximately 320 nm and 500 nm. These peaks correspond to π → π* and n → π* transitions, respectively. The molar absorption coefficients for these transitions fall within the range of 1360–9270 mol^−1^ L cm^−1^, varying based on the solvent used. The peak assigned to the n → π* transition was affected by a large red shift recorded in water over other solvents used (λ_abs_ 517 nm), this might be due to the formation of a strong hydrogen bond between the *CBAPI* sensor and the water molecule. It is clearly shown that the absorption maximum of the *CBAPI* sensor shows nearly no noticeable shift with an increase in polarity of the solvent, but the emission maximum exhibited an appreciable bathochromic shift with an increase in solvent polarities from 390 to 415 nm on exciting at 335 nm, indicating nearly zero dipole moments in the ground state but large dipole moments in the excited state.

**Fig. 3:**
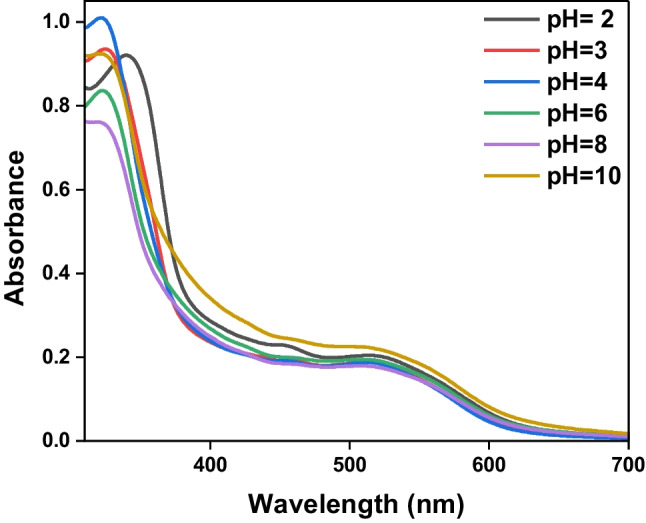
Absorption Spectra of 1 × 10^–4^ mol L.^−1^*CBAPI* sensor at different pH values (pH 2 – pH 10) in DMF/water (0.2:9.8, v/v)

Figure 3 shows the absorption spectra of the CBAPI sensor at different pH values (pH 2 to pH 10) using different buffer solutions. It was noticed that with decreasing pH, the absorption peak assigned to π → π* is greatly shifted towards longer wavelengths (red shift) from λ = 320 nm for pH 10.0 to λ = 340 nm for pH 2.0, while the absorption peak assigned to n → π* is slightly affected by changing pH values. The π-π* peak is indeed pH-dependent, while the n-π* peak shows only minor sensitivity. In an acidic medium, the carbonyl oxygen of the isatin moiety is protonated, affecting the electron density distribution of the CBAPI molecule, and causing changes in the energy levels of the π and π* orbitals. Consequently, the π-π* peak position is altered due to this protonation [36–38].

### Optimizing the Emission Intensity of the CBAPI Sensor for Zn^2+^and Fe^3+^Ions Detection

#### pH Influence

**Fig. 4:**
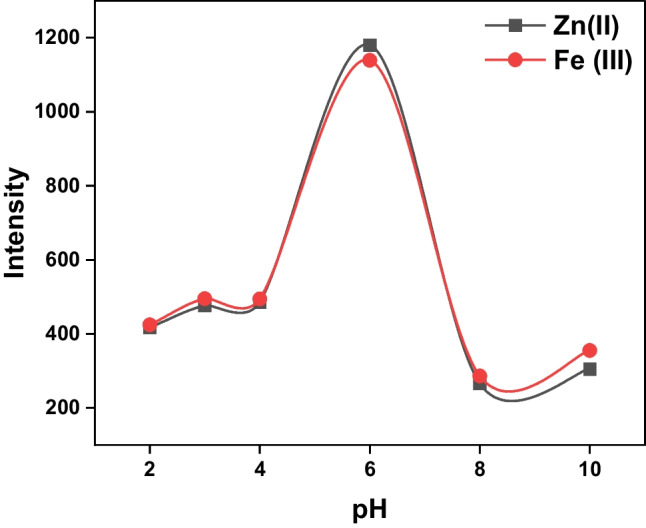
Emission intensity at λ_em_ = 408 nm for detection of Zn (II) and Fe (III) ions as a function of pH using the *CBAPI* sensor

“The impact of varying pH values on the detection of Zn (II) or Fe (III) using the *CBAPI* sensor was investigated across a wide range of pH values (from pH 2 to pH 10) using different buffer solutions (Fig. 4). It was noticed that the higher emission intensity for both metals under study was recorded at pH 6, so further studies were conducted at pH 6 using acetate buffer, which is within an appropriate range for biological and environmental applications. The low intensity at low pH values (pH ≤ 4) and higher pH values (pH ≥ 8) may be due to protonation of imine nitrogen N and carbonyl oxygen C = O of the isatin moiety and precipitation of metal hydroxide, respectively.

#### Method Validation

##### Linearity and Sensitivity

**Fig. 5:**
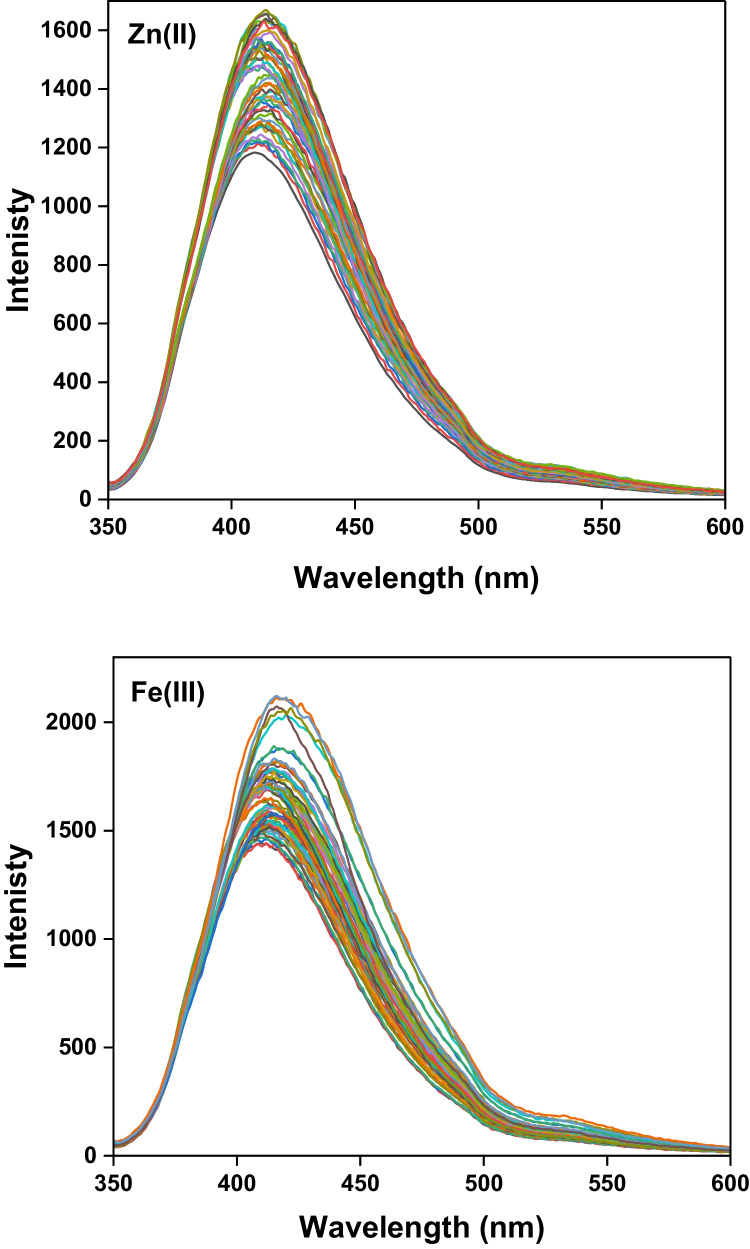
The emission spectra of the *CBAPI* sensor at λ_exc_ 335 nm in acetate buffer (pH = 6.0) with increasing the concentration of Zn^2+^ and Fe^3+^ ions from 3.3 nmol L^−1^ to 80 µmol L.^−1^

The fluorescence titration of different concentrations of metal ions under study (Zn^2+^ or Fe^3+^) to a 20 µmol L^−1^ CBAPI sensor was carried out at pH 6.0 using acetate buffer and at λ_exc_ = 335 nm. Upon the addition of Zn^2+^ ions, there was a notable red shift from the peak at 410 nm to 422 nm, with a remarkable enhancement of emission intensity. Upon the addition of Fe^3+^ ions, a remarkable enhancement of emission intensity is only detected without any shift in wavelength (Fig. 5).

**Fig. 6:**
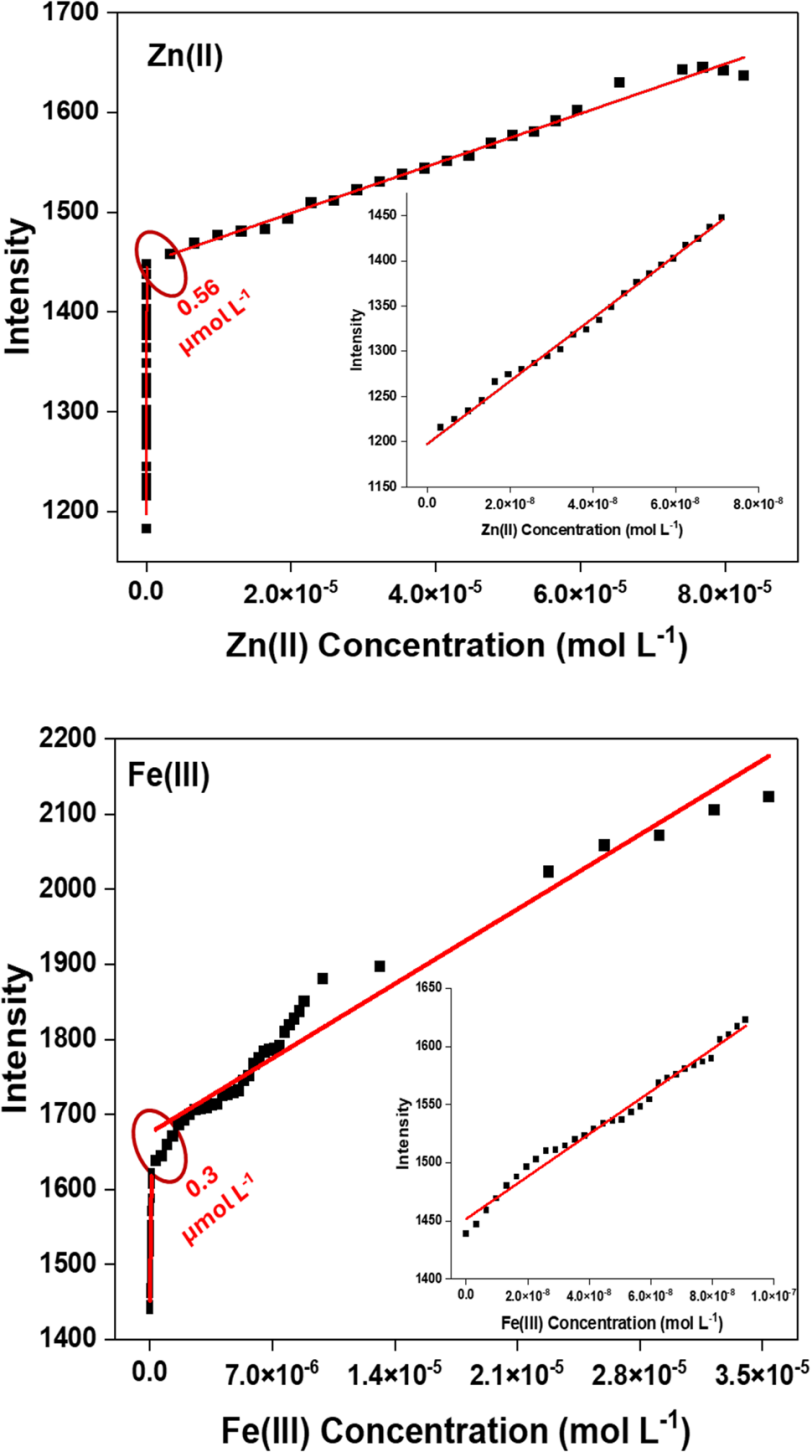
The relationship between the fluorescence intensity of the *CBAPI* sensor and the Zn^2+^ or Fe.^3+^ ions concentration at λ_exc_ 335 nm, λ_em_ 408 nm in acetate buffer (pH = 6.0)

The calibration curve depicted in Fig. 6 showed a linear enhancement in intensity with increasing Zn^2+^ or Fe^3+^ concentrations. The results of the calibration curve for Zn^2+^ determination exhibited two linear ranges: 3.3 to 70 nmol L^−1^ and 3.3 to 80 µmol L^−1^. In the first range, the equation for linear regression is F = 1197.6 + 3.47 × 10^9^ [Zn^2+^] accompanied by a high correlation coefficient of 0.9972, whereas in the subsequent linear range, the linear regression equation is F = 1449.2 + 2.49 × 10^6^ [Zn^2+^] with a correlation coefficient of 0.9888. While the results of the calibration curve for Fe^3+^ determination exhibited two linear ranges: 3.3 to 90 nmol L^−1^ and 0.33 to 35 µmol L^−1^. In the first range, the equation for linear regression is F = 1451.75 + 1082 × 10^9^ [Fe^3+^] with a correlation coefficient of 0.9861, whereas in the second linear range, the linear regression equation is F = 1675.27 + 1.42 × 10^7^ [Fe^3+^] with a correlation coefficient of 0.9591. The *CBAPI* sensor exhibits greater sensitivity to Zn^2^⁺ or Fe^3^⁺ ions, as evidenced by the steeper slope observed in the first linear range compared to the second. The sensitivity of the measurement decreases as the concentration of Zn^2+^ or Fe^3+^ ions increase due to the binding sites of the *CBAPI* sensor being occupied.

The detection limits (*D*_*L*_) (3SD/slope) and quantification limits (*Q*_*L*_) (10SD/slope) were calculated and presented in Table 2. The detection limits for Zn^2+^ or Fe^3+^ ions were 2.90 and 3.59 nmol L^−1^, respectively, and the quantification limits for Zn^2+^ or Fe^3+^ ions were 9.68 and 11.98 nmol L^−1^, respectively. The detection limits for Zn^2+^ or Fe^3+^ ions are compared to those of previously reported sensors **(**Table 3**)**, and the results provide low detection limits over many fluorescent sensors in the literature [39–67]. Furthermore, the World Health Organization (WHO) has defined safe levels of zinc or ferric ions in drinking water as 4.58 × 10⁻^5^ and 5.37 × 10⁻^5^ mol L^−1^, which significantly exceed the levels calculated by the CBAPI sensor [19, 68].

**Table 2:** The correlation coefficients, *D*_*L*_ (detection limit), *Q*_*L*_ (quantitation limit), and *K*_*D*_ (binding constant) for determination of Zn^2+^ or Fe^3+^ ions using the *CBAPI* sensor at λ_em_ 408 nm, λ_exc_ 335 nm and at pH 6.0

Parameter	Zn (II) ion	Fe (III) ion
D_L_ (nmol L^−1^)	2.90	3.59
Q_L_ (μmol L^−1^)	9.68	11.98
Regression coefficient	0.9972	0.9861
Slope	3.47 × 10^9^	1.82 × 10^9^
Intercept	1.20 × 10^3^	1.45 × 10^3^
Standard deviation	3.36 (n = 24)	2.18 (n = 31)
Linearity range (nM)	3.3—70	3.3—90
Binding Constant K_D_ (M^−1^)	1.77 × 10^7^	6.41 × 10^6^

**Table 3:** Comparison between the *CBAPI* sensor with the literature-reported methods for Zn^2+^ and Fe^3+^ ions detection

Sensor	Detection limit (nmol L^−1^)	Reference
Detection limits of Zn (II) ions		
N,N′-phenylenebis(salicylideaminato)	1.5 × 10^2^ nM	[39]
2-(benzo[d]thiazol-2-yl) phenol (BTP)	23.6 nM	[40]
2-(((pyridin-2-ylmethyl)imino)methyl)phenol	62 nM	[41]
di-2-picolylamine-dithiolcarbamate (DPA-DTC)/proline-dithiolcarbamate (P-DTC)	700 nM	[42]
Fluorescent quantum dots (QDs) as a promising alternative for organic dyes CdTe QDs	1.2 × 10^3^ nM	[43]
3-(benzo[d]thiazol-2-yl)-4-hydroxy-2H-chromen-2-one	35.8 nM	[44]
(E)-N’-(5-allyl-2-hydroxy-3-methoxybenzylidene) nicotinohydrazide	4.35 nM	[45]
tert-butyl (2-bromoethyl)carbamate (4)	107 nM	[46]
tetraphenylporphyrin β-cyclodextrin	5.0 × 10^2^ nM	[47]
The Schiff-base ligand	95.3 nM	[48]
TYMN ((E)-1-((thiazol-2-ylimino)methyl)naphthalen-2-ol)	31.1 nM	[49]
(L, Dansyl-Asp-His-NH_2_)	36.8 nM	[50]
1-[[(2-furanylmethyl)imino]methyl]-2-hydroxyjulolidine	7.50 × 10^3^ nM	[51]
Methyl 2-((7-hydroxy-4-methyl-2-oxo-2H-chromen-8-yl)methyleneamino)-3-phenyl- propanoate	3.43 nM	[52]
2,20 -(1E,10 E)-(2,2-azanediylbis(ethane2,1-diyl)bis(azan -1-yl-1-ylidene))bis(methan-1-yl-1-ylidene) dipnenol, TAS	83 nM	[53]
*CBAPI* sensor	2.90 nM	Present study
Detection limits of Fe (III) ions		
2-azido-1-ethanol, 910-bis(bromo- methyl)anthracene, 2-(prop-2-yn-1-yloxy)naphthalene	3.14 × 10^2^ nM	[54]
Rhodamine-based probe (RC)	14 nM	[55]
Fluorescent probe Rh-2 based on bis- (rhodamine)	12.4 nM	[56]
Polyethyleneimine (PEI)–modified reduced graphene oxide (rGO)	1.12 × 10^3^ nM	[57]
Porous copper nanoclusters (p-Cu NCs)	23.4 nM	[58]
((E)-2-((4-(diethylamino)benzylidene)amino)benzoic acid, DBAB)	21.7 nM	[59]
Fluorescein isothiocyanate (FITC)	4.60 × 10^3^ nM	[60]
Carbon quantum dots (CQDs)	1.37 × 10^4^ nM	[61]
Fluorescent carbon nanoparticles (CNPs) by using dopamine	3.20 × 10^2^ nM	[62]
Dual-emission carbon dots DCDs	8.0 × 10^2^ nM	[63]
Dual-response fluorescent probe (QLBM) based on quinoline and benzimidazole groups	1.24 × 10^2^ nM	[64]
Water soluble carbon nanoparticles (CNPs)	3.21 × 10^5^ nM	[65]
Fluorescence resonance energy transfer (FRET)	2.54 × 10^3^ nM	[66]
4,4′-(thiazolo[5,4-d]thiazole-2,5-diyl)bis(N,N-bis(4-methoxyphenyl)aniline) (TTz-1)	0.29 × 10^2^ nM	[67]
*CBAPI* sensor	3.59 nM	Present study

##### Method Selectivity

**Fig. 7:**
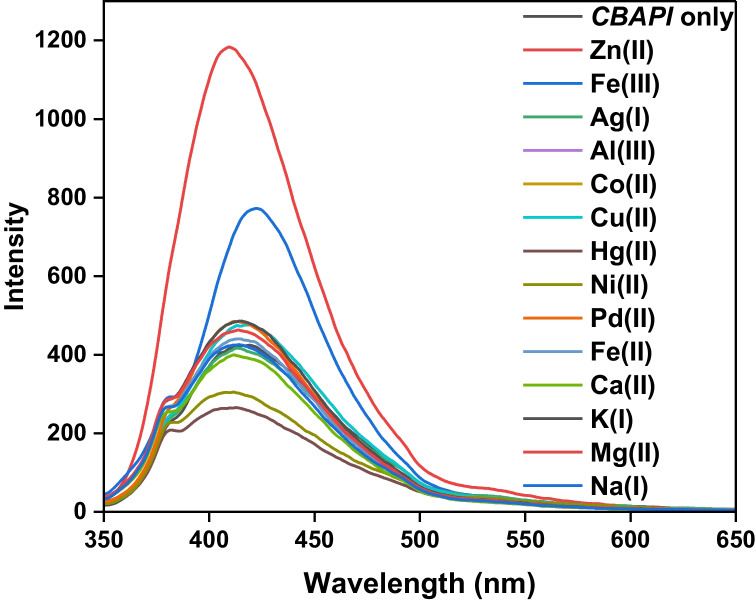
The emission spectra of the *CBAPI* sensor at pH = 6.0 in the presence of different metal ions at λ_exc_ 335 nm

**Fig. 8:**
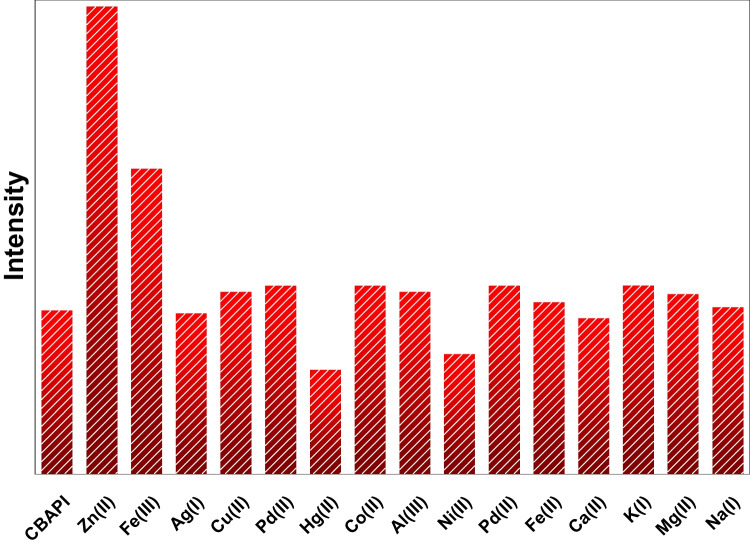
Intensity at λ_em_ 408 nm of *CBAPI* sensor in presence of 10 μmol L^−1^ different metal ions at λ_ex_ 335 nm and pH 6.0

The selectivity of the *CBAPI* sensor to other metal ions was separately investigated under optimum conditions. The emission spectrum of the 20 µmol L^−1^*CBAPI* sensor in DMF/water (0.2:9.8, v/v) at pH 6.0 and λ_exc_ 335 nm was monitored in the presence of 20 µmol L^−1^ of interfering ions (Ag^+^, Al^3+^, Cu^2+^, Co^2+^, Hg^2+^, Ni^2+^, and Pd^2+^) (Fig. 7), and the emission intensity at 408 nm was recorded (Fig. 8). The results showed that all cations displayed an insignificant change in the emission spectra of the *CBAPI* sensor.

#### Molar Ratios and Binding Constants

To further demonstrate the stoichiometry between the *CBAPI* sensor and Zn (II) or Fe (III) ions, the molar ratio method was used, as shown in SI: Fig. 3. Emission intensities at λ_em_ = 408 nm and at pH 6.0 are measured when using different concentrations of the *CBAPI* sensor (from 1 × 10^–5^ mol L^−1^ to 4.5 × 10^–5^ mol L^−1^), while the concentration of metal ions under study is kept constant (1 × 10^–5^ mol L^−1^). The [*CBAPI*]/[M] ratio was simultaneously plotted against the fluorometric intensity for each complex, the results showed 2:1 and 3:1 (*CBAPI*-to-metal) ratios for Zn/*CBAPI* complex and Fe/*CBAPI* complex, respectively.

The Benesi-Hildebrand equation (***Eq. ***1) was used to determine the binding constants (*K*_*D*_) between the *CBAPI* sensor and the metal ions (Zn (II) or Fe (III)) under investigation [69]. *K*_*D*_ (unit of liter per mole) is derived from the quotient of y-intercept and slope. Where *F*_*L*_ is the limiting intensity of fluorescence, and [M^n+^] is the concentration of Zn (II) or Fe (III) ions. *F*^*o*^ and *F* represent the emission intensity at λ_em_ = 408 nm and at pH 6.0 in the presence and absence of metal ions under study, respectively.

1 $$\frac{F^\circ }{F-F^\circ }= \propto + \frac{\propto }{{K}_{D} \left[{M}^{n+}\right]}, \propto = \frac{1}{{F}_{L}-F^\circ }$$

A plot of F^o^/(F-F^o^) versus 1/[M^n+^] gave a linear relationship (SI: Fig. 4) where the concentration of the *CBAPI* sensor was kept constant (2 × 10^–5^ mol L^−1^), while the concentration of metal ions under study (Zn (II) or Fe (III)) set up from 3.3 nmol L^−1^ to 80 µmol L^−1^. Using this calculation method, the binding constants *K*_*D*_ for Zn/*CBAPI* and Fe/*CBAPI* complexes were determined to be 1.77 × 10^7^ and 6.41 × 10^6^ M^−1^ at pH 6.0.

### Mechanism of Fluorescence Sensitization

The binding mechanism involves the coordination of Zn^2^⁺ or Fe^3+^ ions with the imine nitrogen (azomethine) and carbonyl oxygen of the ketone group in the isatin moiety. This coordination leads to a remarkable enhancement of the emission intensity at 408 nm in the *CBAPI* sensor, making it valuable for analytical and biological applications in detecting Zn^2^⁺ or Fe^3+^ ions (SI: Scheme 1). Additionally, the chelation to Zn^2^⁺ or Fe^3+^ ions induces rigidity in the formed complexes, thereby increasing the Chelation Enhanced Fluorescence (CHEF) effect [70].

### Application to Human Hair Samples

To further confirm the quantification ability of the *CBAPI* sensor to detect Zn^2+^ ions, The synthesized (2.0 × 10^–5^ mol L^−1^) *CBAPI* sensor was applied to determine the concentrations of Zn in two human hair samples at pH 6.0. The extracted Zn^2+^ ions were analyzed using Perkin Elmer Analyst 100 Atomic Absorption Spectroscopy (AAS), and the results were compared with the results obtained when using the *CBAPI* sensor. As shown in Table 4, the results of two hair samples were in good agreement with AAS results, which shows that the *CBAPI* sensor is an effective and accurate sensor for detecting Zn in human hair elements.

**Table 4:** Concentrations (ppm) of Zn^2+^ ions in human hair

Sample	Concentration of proposed method (ppm)	
	AAS	*CBAPI* sensor
Sample No. 1	0.355	0.348
Sample No. 2	0.367	0.363

### Assessing Analytical GREEnness Metric Using CBAPI Sensor

**Fig. 9:**
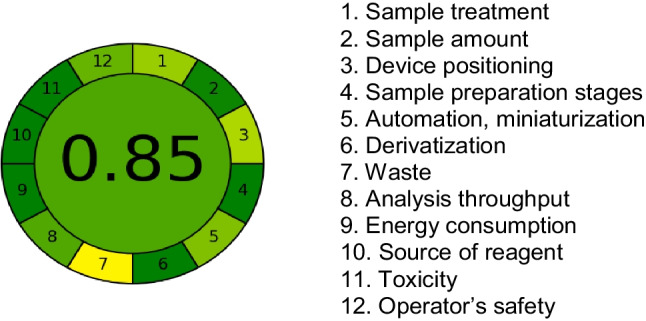
AGREE Assessment for detecting of Zn^2+^ or Fe^3+^ ions using *CBAPI* sensor

The reliability of the greenness attributes of the spectrofluorimetric method for detecting Zn^2+^ or Fe^3+^ ions using the *CBAPI* sensor was evaluated through the Evaluation of Greenness Attributes [71, 72]. The synthesized sensor received an AGREE score of 0.85, reflecting the high greenness attributes of the fluorometric detection of Zn^2+^ or Fe^3+^ ions based on the *CBAPI* sensor. This high score (0.85) is primarily attributed to the detection method carried out in DMF/water (0.2:9.8, v/v) at a pH near the physiological value (pH 6.0), without the use of toxic solvents even during the extraction and preconcentration procedures emphasizing the eco-friendly nature of this approach (Fig. 9).

## Conclusion

The *CBAPI* sensor was synthesized and characterized using FT-IR and ^1^H-NMR spectroscopy. Its spectroscopic parameters in different solvents were determined. The *CBAPI* sensor, with its remarkable sensitivity and selectivity, has emerged as a valuable tool for detecting Zn^2^⁺ and Fe^3^⁺ ions, and its performance was compared with the AAS technique for zinc detection in human hair samples. By calculating detection and quantification limits through fluorescence titration under optimum conditions, we demonstrated the strong binding affinity between Zn^2^⁺ or Fe^3^⁺ ions and the *CBAPI* sensor, as well as its high selectivity over other interfering cations. Finally, the proposed method achieved a high AGREE score, emphasizing its eco-friendly nature.

## Supplementary Information

Below is the link to the electronic supplementary material.

Supplementary file1 (DOCX 537 KB)

## Data Availability

Not applicable.
