# Supplementary material for: Eco-friendly Fluorescent Sensor for Sensitive and Selective Detection of Zn2+ and Fe3+ Ions: Applications in Human Hair Samples
Source: J Fluoresc. 2024 Jul 3;35(6):4213–24. doi: 10.1007/s10895-024-03798-3 (PMC12206213; doi:10.1007/s10895-024-03798-3)
Supplement: Supplementary file 1 — Supplementary file1 (DOCX 537 KB) [file 10895_2024_3798_MOESM1_ESM.docx]

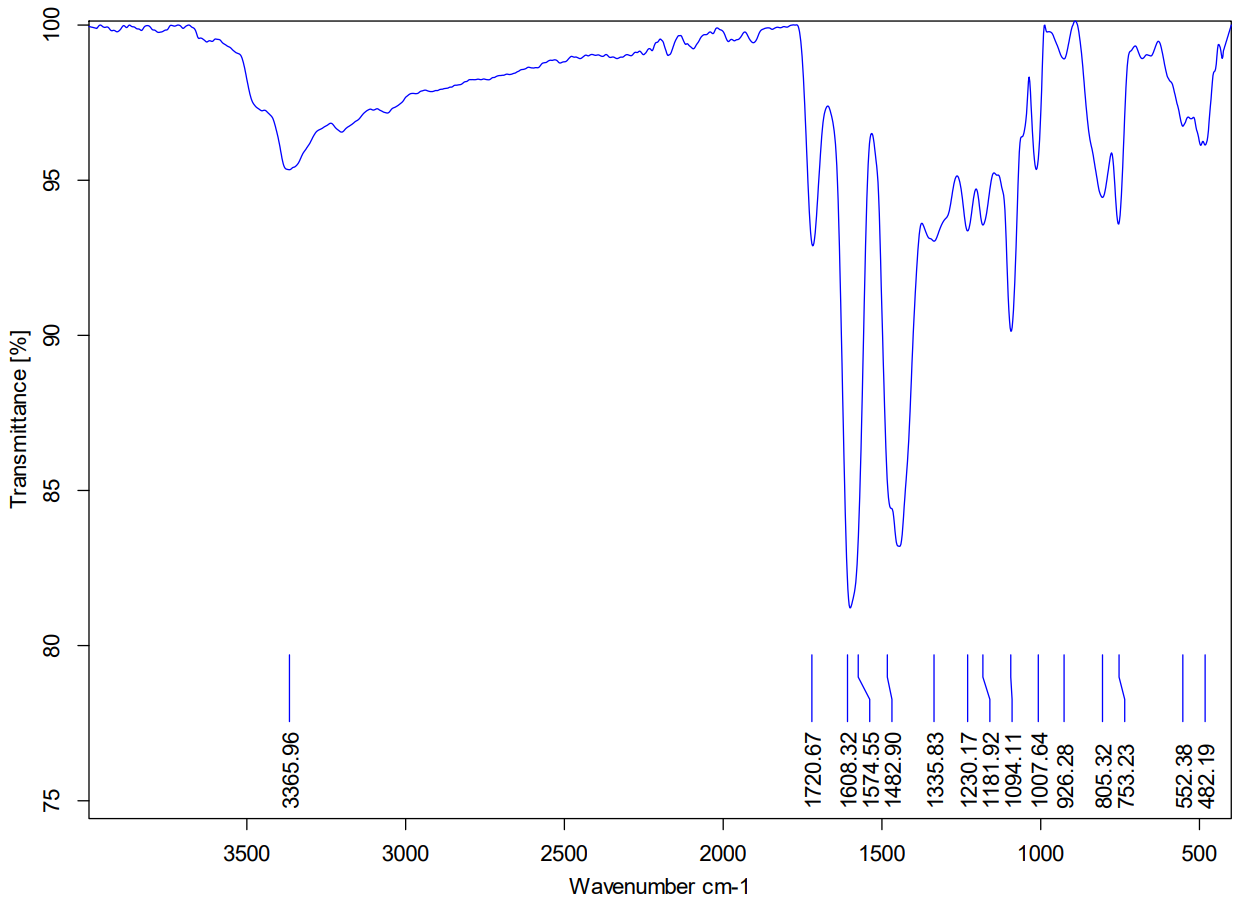


**SI: Fig. 1:** FT-IR spectrum of the *CBAPI* sensor


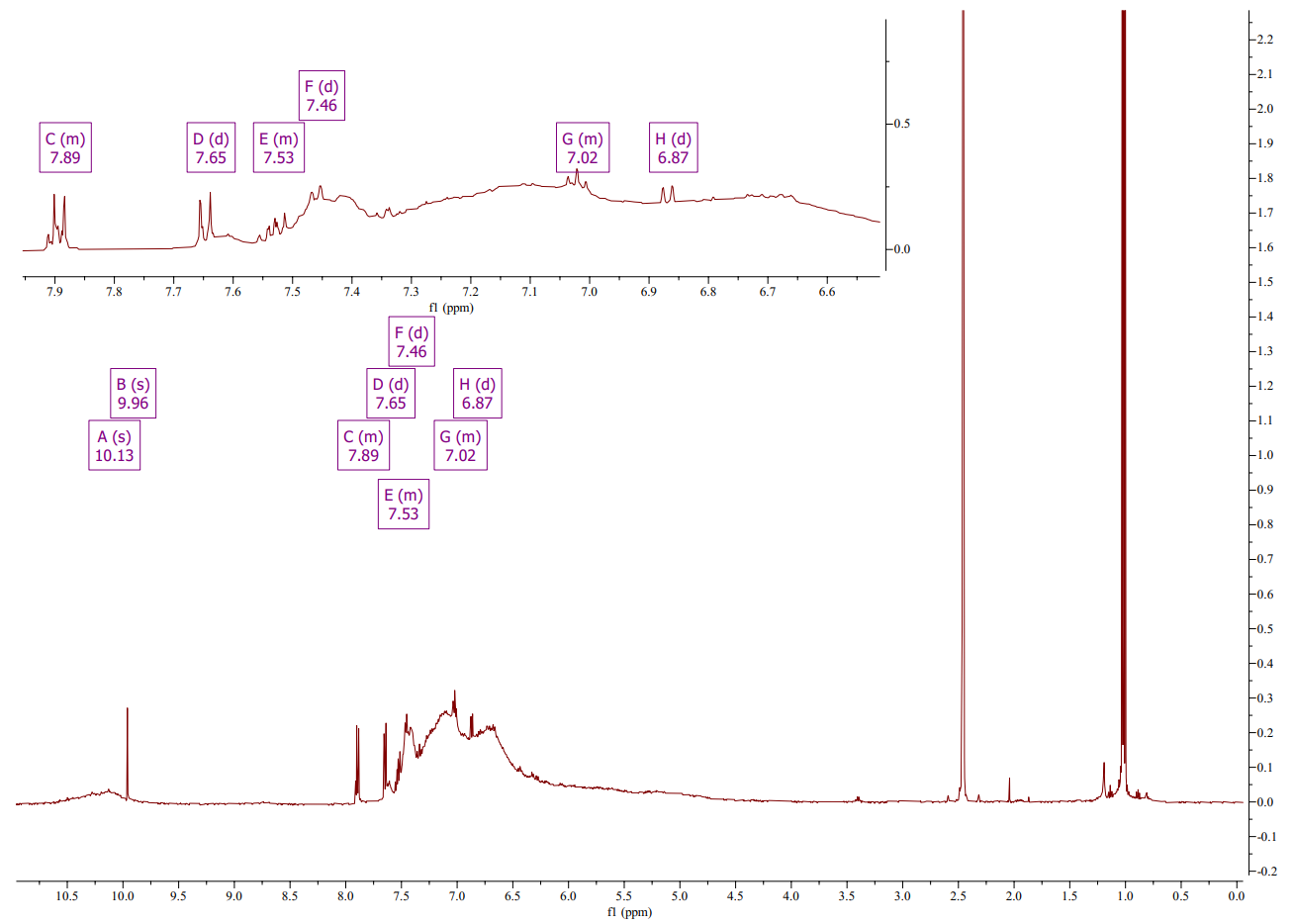


**SI: Fig. 2:** ^1^H-NMR spectrum of the *CBAPI* sensor

**SI: Fig. 3:** Molar ratio plot for Zn/*CBAPI* and Fe/*CBAPI* complexes at λ_ex_= 335 nm, λ_em_= 408 nm and at pH 6.0.

**SI: Fig. 4:** Benesi-Hildebrand relation for the interaction of different concentrations of Zn^2+^ or Fe^3+^ ions with the *CBAPI* sensor at λ_exc_ 335 nm, λ_em_ 408 nm in acetate buffer (pH = 6.0).

**SI: Scheme 1:** Predicted structures of Zn/CBAPI and Fe/CBAPI complexes

**Instrumental Section**

Buffer solutions (pH 2.0, 4.0, 6.0, 8.0, 10.0 and 12.0) was prepared using (KCl- HCl), (sodium acetate tri-hydrate - acetic acid), (HCl - disodium hydrogen phosphate), (NaOH - disodium hydrogen phosphate), and (KCl- NaOH) respectively.

The infrared spectra were recorded on a Bruker ALPHA II FT-IR spectrophotometer using the potassium bromide disc technique. Proton magnetic resonance ^1^H NMR spectra were recorded on a Bruker 400 MHz-NMR spectrometer with DMSO-d_6_ as the solvent.


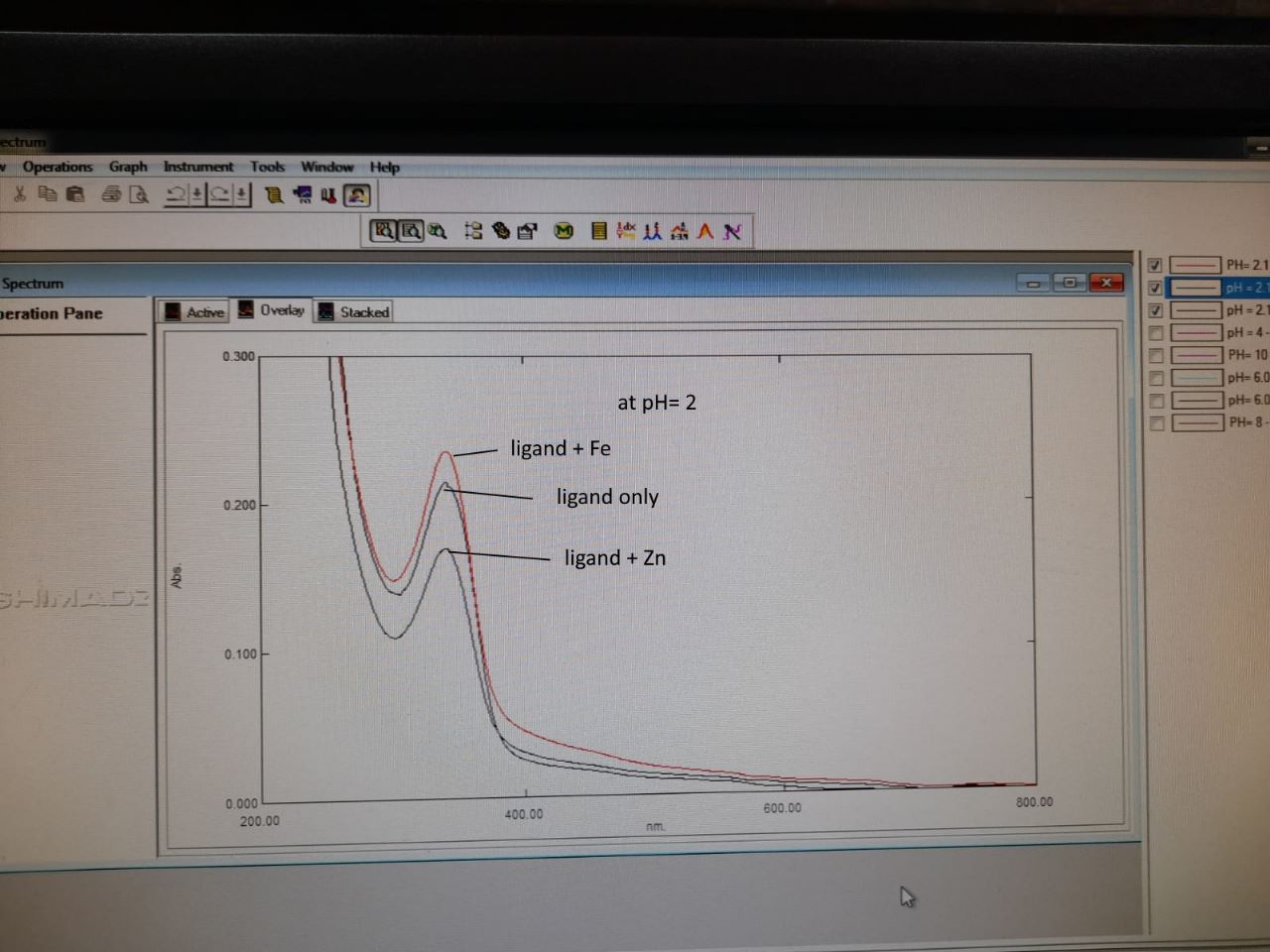


The absorption spectrum of 2x10^-5^ mol/L CBAPI only and in presence of 1 x10^-5^ mol /L Fe(III) or Zn(II) ions at pH 6.0.
